# HIV Evolution in Early Infection: Selection Pressures, Patterns of Insertion and Deletion, and the Impact of APOBEC

**DOI:** 10.1371/journal.ppat.1000414

**Published:** 2009-05-08

**Authors:** Natasha Wood, Tanmoy Bhattacharya, Brandon F. Keele, Elena Giorgi, Michael Liu, Brian Gaschen, Marcus Daniels, Guido Ferrari, Barton F. Haynes, Andrew McMichael, George M. Shaw, Beatrice H. Hahn, Bette Korber, Cathal Seoighe

**Affiliations:** 1 Institute of Infectious Disease and Molecular Medicine, University of Cape Town, Observatory, Cape Town, South Africa; 2 Centre for High-Performance Computing, CSIR Campus, Rosebank, Cape Town, South Africa; 3 Theoretical Division, Los Alamos National Laboratory, Los Alamos, New Mexico, United States of America; 4 Santa Fe Institute, Santa Fe, New Mexico, United States of America; 5 University of Alabama at Birmingham, Birmingham, Alabama, United States of America; 6 Department of Mathematics and Statistics, University of Massachusetts, Amherst, Massachusetts, United States of America; 7 Weatherall Institute of Molecular Medicine, University of Oxford, John Radcliffe Hospital, Oxford, United Kingdom; 8 Duke University, Durham, North Carolina, United States of America; 9 School of Mathematics, Statistics and Applied Mathematics, National University of Ireland Galway, Galway, Ireland; NIH/NIAID, United States of America

## Abstract

The pattern of viral diversification in newly infected individuals provides information about the host environment and immune responses typically experienced by the newly transmitted virus. For example, sites that tend to evolve rapidly across multiple early-infection patients could be involved in enabling escape from common early immune responses, could represent adaptation for rapid growth in a newly infected host, or could represent reversion from less fit forms of the virus that were selected for immune escape in previous hosts. Here we investigated the diversification of HIV-1 *env* coding sequences in 81 very early B subtype infections previously shown to have resulted from transmission or expansion of single viruses (n = 78) or two closely related viruses (n = 3). In these cases, the sequence of the infecting virus can be estimated accurately, enabling inference of both the direction of substitutions as well as distinction between insertion and deletion events. By integrating information across multiple acutely infected hosts, we find evidence of adaptive evolution of HIV-1 *env* and identify a subset of codon sites that diversified more rapidly than can be explained by a model of neutral evolution. Of 24 such rapidly diversifying sites, 14 were either i) clustered and embedded in CTL epitopes that were verified experimentally or predicted based on the individual's HLA or ii) in a nucleotide context indicative of APOBEC-mediated G-to-A substitutions, despite having excluded heavily hypermutated sequences prior to the analysis. In several cases, a rapidly evolving site was embedded both in an APOBEC motif and in a CTL epitope, suggesting that APOBEC may facilitate early immune escape. Ten rapidly diversifying sites could not be explained by CTL escape or APOBEC hypermutation, including the most frequently mutated site, in the fusion peptide of gp41. We also examined the distribution, extent, and sequence context of insertions and deletions, and we provide evidence that the length variation seen in hypervariable loop regions of the envelope glycoprotein is a consequence of selection and not of mutational hotspots. Our results provide a detailed view of the process of diversification of HIV-1 following transmission, highlighting the role of CTL escape and hypermutation in shaping viral evolution during the establishment of new infections.

## Author Summary

HIV is a rapidly evolving virus, displaying enormous genetic diversity between and even within infected individuals, with implications for vaccine design and drug treatment. Yet, recent research has shown that most new infections result from transmission of a single virus resulting in a homogeneous viral population in early infection. The process of diversification from the transmitted virus provides information about the selection pressures experienced by the virus during the establishment of a new infection. In this paper, we studied early diversification of the *envelope* gene in a cohort of 81 subjects acutely infected with HIV-1 subtype B and found evidence of adaptive evolution, with a proportion of sites that tended to diversify more rapidly than expected under a model of neutral evolution. Several of these rapidly diversifying sites facilitate escape from early cytotoxic immune responses. Interestingly, hypermutation of the virus, brought about by host proteins as a strategy to restrict infection, appeared to be associated with early immune escape. In addition to single base substitutions, insertions and deletions are an important aspect of HIV evolution. We show that insertion and deletion mutations occur evenly across the gene, but are preferentially fixed in the variable loop regions.

## Introduction

Adaptation of HIV-1 to new hosts, in particular to the initial immune response, is likely to influence the establishment and progression of HIV infection. Mutations that enable escape from the host's immune responses will come under selective pressure, in an order that reflects the timing of the immune responses, underlying mutational rates, and the relative fitness costs of mutations. In addition, escape mutations that occurred in the infecting host, particularly those resulting in a reduction of viral fitness [1], will come under selective pressure to revert to wild-type in the newly infected individual [2]. If transmission itself is a selective process, such that, for example, viral variants that are adapted to replication in mucosal cell types are selectively transmitted, the virus may experience selection to acquire forms that are adapted to replication in other host tissues. Since the viral envelope glycoprotein (Env) plays a vital role in transmission, is an early target of immune responses, and a key determinant of target cell tropism, the early evolution of the HIV-1 *envelope* gene is of particular interest.

Env is a trimeric glycoprotein composed of three gp120 and gp41 heterodimers that are exposed on the viral surface and are anchored on the viral particle by the transmembrane domains of the gp41 subunit. Viral cell entry is initiated by the binding of gp120 to the CD4 receptor, followed by CCR5 or CXCR4 coreceptor binding [3], which then induces conformational changes in gp120 that trigger the activation of gp41 [4]–[6]. Activated gp41 leads to fusion with the target cell membranes, and virus entry [5],[7]. The development of vaccines capable of eliciting antibodies against Env that can block viral entry is complicated by the vast sequence variability between viruses, escape variants arising through the high mutation and recombination rates coupled with immune selection, as well as by the large number of N-linked glycans in gp120 that provide the virus with a protective coat [5],[8]. In addition, gp120 consists of five highly variable (V1–V5) regions interspersed among more conserved (C1–C5) regions [9]; of these, V1, V2, V4 and V5 are notable for rapid shifts in the length, number and localization of glycosylation sites [10],[11]. The variable loops shield more conserved receptor binding regions [12], and only when the CD4-gp120 binding takes place, do structural changes expose previously masked epitopes and surfaces [13],[14].

The transmission of HIV is associated with a severe population bottleneck, such that, in most cases, new infections result from transmission or expansion of a single virus [15]. We have recently shown that the sequence of the virus that is transmitted can be identified accurately through direct sequencing of the uncloned DNA amplicons derived from single genome amplification (SGA) of plasma viral RNA sampled during acute and early infection [15],[16]. For that study, each of 102 HIV-1 subtype B primary infections was classified into one of six clinical stages according to patterns of laboratory assay reactivity defined by Fiebig *et al.* [17]. Out of 102 patients, there was evidence of infection by or rapid outgrowth of a single virus (or in three cases possibly two closely related viruses) for 81 of these individuals [15]. In the present study we have carried out a detailed analysis of the early evolution and divergence of the virus from the ancestral infecting strain in these 81 homogeneous infections. We restricted our study to the homogeneous infections because in these patients the observed sequence diversity is expected to be due mainly, or entirely (in the case of transmission of a single viral variant), to mutations that have occurred following transmission. The data analyzed in this study consisted of sets of between 10 and 67 sequences per patient, generated previously [15] using SGA from single time-points in acute or early infection.

Phylogenetic trees of the sequences from individual patients had short branch lengths and few internal branches, reflecting the short time since transmission and rapid expansion in the new host. It was not possible to apply standard phylogenetic models of evolution to these data to assess whether diversification of the virus following transmission is primarily a stochastic process, or driven by evolutionary selection pressure, because sequences from individual patients provide too little information to allow statistical discrimination between alternative evolutionary models. However, by considering all patients simultaneously, it was possible to determine whether similar patterns of sequence diversification were repeated either in multiple patients or within the same patient. Shared patterns of rapid diversification at the same codons in early infection can reveal sites that are typically involved in viral adaptation to the new host at the earliest stage of infection. In cases where these sites are associated with specific host immune responses, they shed light on the nature, extent, and timing of the immune responses that most frequently exert selective pressure on the transmitted virus during acute and early infection. However, there is also the possibility that shared mutations across multiple patients may be the result of deviation from the expected mutation rates, so that particular sites with high mutation rates, for example resulting from APOBEC-mediated substitutions, might be mutated in an unexpectedly high proportion of patients.

Using HyPhy [18], an interpreted computer programming environment for fitting evolutionary models, we have developed a method that can identify sites in *env* that tend to diversify most rapidly across recently infected patients, and explored the reasons for rapid diversification at these sites. The sequence of the transmitted founder virus at or near the time of transmission, which had been inferred previously [15], enabled us to determine the direction of single base mutations and to distinguish between insertions and deletions. A relative reduction in the length of the variable loops of *env* has previously been reported at transmission compared to chronic infection [19],[20] and this is thought to be an important characteristic of acute subtype A and C infections [21]. Indels may also affect antibody recognition [22], and, in consequence, may be under selective pressure. We have also carried out a detailed analysis of the length distribution and locations of insertions and deletions in early infection and investigated the existence of indel hotspots in the *envelope* gene. In this study we found that i) there is evidence of positive selection in early HIV-1 infection, which appears to be driven in many cases by escape from early cytotoxic T lymphocyte (CTL) responses; ii) in several cases CTL escape occurred via mutations in the APOBEC sequence context, suggesting a role for APOBEC in determining the pathway of immune escape; and iii) frameshifting insertions and deletions are evenly spread throughout *env* but are preferentially fixed in the variable regions. These results provide the most detailed view yet of the process of diversification of the homogeneous viral population following transmission.

## Results

### Model-based inference of selection in early infection

We fitted models of codon sequence evolution to 2,207 *env* coding sequences from early stages of HIV-1 infection as described in the Methods section. In order to accumulate evidence of selection from multiple patients the models were implemented such that at a given site the value of ω (the ratio of nonsynonymous to synonymous substitution rates) was constrained to be the same in all patients. This enabled us to use standard model comparison techniques to investigate whether there was evidence that the mean value of ω across patients was significantly greater than one at a subset of sites in the alignment (see Methods). We found that, as expected, the majority of codon sites evolve under the influence of purifying selection (with ω<1) in early infection and the mean value of ω across all sites in all patients was 0.67. Three overlapping groups of patients were considered, consisting of individuals in the earliest clinical stages (Fiebig stages I and II), earliest through intermediate stages (Fiebig stages I through III) and all patients in early infection (Fiebig stages I through V). For the data from Fiebig stages I and II the model allowing a subset of sites to evolve under positive selection did not provide a significantly better fit to the data than the model in which positive selection was not permitted (p = 0.52; Table 1). For the other two datasets, however, the selection model (M2a) provided a significantly better fit to the data than the model in which selection was excluded (M1a). M1a was rejected against M2a with p-values 0.006 and 0.0001 in the case of the datasets from Fiebig stages I–III and stages I–V, respectively (Table 1).

**Table 1:** Parameter estimates for the neutral and selection model applied to *env* sequences from different Fiebig stage datasets.

Fiebig Stage	dN/dS: Neutral Model (M1a)	dN/dS: Selection Model (M2a)	2 * Delta Log Likelihood	P-value (M1a vs M2a)
I–II	0.6912	0.6995	1.2833	0.5264
I–III	0.6687	0.7016	10.0741	0.0065
I–V	0.6307	0.7130	18.3186	0.0001

For this analysis all individual sequences with evidence of APOBEC-mediated hypermutation were removed using Hypermut 2.0 (www.hiv.lanl.gov). For fifteen of the patients the *env* sequences showed an overall elevated rate of G→A mutations in a sequence context associated with APOBEC hypermutation (using the methodology described in the supplementary data of [15]; see Figure S1) even though no individual sequences showed evidence of hypermutation Hotspots of mutation could create false positive signatures of positive selection, particularly if the same hotspots are repeated across multiple patients, as we would expect for mutation hotspots resulting from APOBEC hypermutation. To limit the impact of hypermutation on our results, these patients were removed from the dataset, leaving 66 subjects. Interestingly, even with these precautions, we found that sites that were placed in the set of sites with ω>1 (positive selection site class) are enriched for the APOBEC-motif (see Table 2). Because of the potential biological relevance of these mutations we performed a second analysis including all 81 homogeneous patients, bearing in mind the caveat that the model may be affected by the higher rate of G→A mutation in the APOBEC context and that some of the sites identified from this dataset may reflect mutational hotspots rather than selection acting on the amino acid sequence.

**Table 2:** Positive selection results obtained using HyPhy from a dataset excluding individuals with sequences enriched for APOBEC hypermutation, as well as from the complete dataset including the hypermutated sequences (the latter sites are indicated with +).

HXB2	Data	Location	Posterior Probability	p-value/ q-value	Number of subjects with variation	APOBEC3 in subject's mutations	Timing (Fiebig Stage)	Number of subjects with a mutational pattern out of all 81 subjects	CTL testing
62	79	gp120 C1	0.765	-	5	4 of 5	I–V	2 D to N, 1 D to Y, 2 E to K	Yes
64	81	gp120 C1	0.505	-	2	No	I–V	*2 E to G*	Yes
66	83	gp120 C1	0.979	-	4	No	I–V	*4 H to Y*	Yes
66 ∼	83	gp120 C1	-	*0.006/0.04*	4	No	IV–VI	*4 To Y*	Yes
66 ∼	83	gp120 C1	-	*0.006/0.05*	4	No	IV–VI	*4 Away from H*	Yes
175	226	gp120 V2	0.983	-	7	No	I–V	3 L to P, 3 L to F, 1 N to S	No
176	227	gp120 V2	0.655	-	2	No	I–V	1 F to S, 1 F to V	No
232	306	gp120 C2	0.853	-	4	No	I–V	1 K to E, 1 K to R, 1 T to A, 1 T to M	No
242	316	gp120 C2	0.652	-	3	No	I–V	2 V to I, 1 V to L	No
274	349	gp120 C2	0.722	-	3	No	I–V	1 S to T, 1 S to F, 1 S to P	No
322 +	396	gp120 V3	0.51	-	4	2 of 4	I–V	2 E to K, 1 E to G, 1D to N	No
337	412*	gp120 C3	0.932	-	5	2 of 5	I–V	1 K to E, 1 K to R, 1 E to K, 1 D to N, *1 D to A*	No
344 +	419	gp120 C3	0.588	-	3	No	I–V	*1 K to M*, 1 Q to L, 1 R to G	No
347 +	422	gp120 C3	0.91	-	5	4 of 5	I–V	2 R to K, 1 K to R, 1 G to R, *1 G to E*	No
354	431	gp120 C3	0.895	-	3	2 of 3	I–V	1 E to K, 1 G to E, *1 K to R/T*	Yes
360	439	gp120 C3	0.897	-	3	No	I–V	1 V to G, 1 V to A, *1V to G/A*	Yes
372 ∼	454*	gp120 C3	-	*0.070/0.14*	1	No	I–II*	*1 Away from A*	No
381 ∼	463	gp120 C3	-	*0.042/0.19*	4	4 of 4	III–VI	4 To K	No
381 ∼	463	gp120 C3	-	*0.042/0.09*	4	4 of 4	III–VI	4 Away from E	No
460	566	gp120 C4	0.87	-	2	No	I–V	1 N to K, *1 D to N/K/T*	Yes
460 ∼	566	gp120 C4	-	*0.006/0.08*	2	No	IV–VI	*2 Away from N*	Yes
482 +	601	gp120 C5	0.669	-	5	5 of 5	I–V	*5 E to K*	No
509	634	gp120 C5	0.904	-	7	7 of 7**	I–V	7 E to K	No
513	638	gp41	0.728	-	2	No	I–V	1 V to S, 1 V to G	No
518	646	gp41	0.999	-	9	No	I–V	2 M to I, 7 M to V	No
587	719	gp41	0.77	-	2	No	I–V	2 L to I	No
588	720	gp41	0.546	-	3	2 of 3	I–V	2 R to K, 1 K to R	No
612	744	gp41	0.841	-	2	No	I–V	*1 I to T/S/N*, 1 T to I	Yes
632 ∼	768	gp41	-	*0.042/0.10*	4	4 of 4	III–VI	4 To K	No
632 ∼	768	gp41	-	*0.037/0.17*	4	4 of 4	III–VI	4 Away from E	No
648 ∼	786	gp41	-	*0.080/0.18*	3	3 of 3	III–VI	3 To K	No
651	789	gp41	0.598	-	3	No	I–V	*2 N to S*, 1 S to G	No
696	834	gp41	0.635	-	7	6 of 7	I–V	*6 R to K*, 1 R to S	No
700	838*	gp41	0.535	-	3	No	I–V	*1 A to V*, 1 A to T,1 T to S	No
702 +	840	gp41	0.533	-	3	No	I–V	2 L to F, 1 L to P	No
703 +	841	gp41	0.541	-	3	No	I–V	1 S to A, 1 S to F, 1 S to P	No
817 ∼	965	gp41	-	*0.002/0.29*	2	No	V–VI	*2 To T*	Yes
817 ∼	965	gp41	-	*0.003/0.048*	2	No	V–VI	*2 Away from A*	Yes
831	979	gp41	0.664	-	2	2 of 2	I–V	2 E to K***	Yes
831 ∼	979	gp41	-	*0.029/0.082*	2	2 of 2	V–VI	*2 To K*	Yes
833	981	gp41	0.993	-	2	No	I–V	1 V to A, *1 V to A/G*	Yes
833 ∼	981	gp41	-	*0.010/0.15*	2	No	V–VI	*2 To V*	Yes
841	989	gp41	0.772	-	4	No	I–V	1 L to H or gap, 1 L to P, 1 L to F, 1 I to T	Yes

Sites identified using the maximum likelihood phylogeny-based method are indicated with ∼ . The location, timing, and mutational patterns observed are provided as well as an indication of whether CTL testing was carried out or not.

**Key for** **Table 2**

**HXB2:** Coordinates listed according to HXB2 numbering (http://www.hiv.lanl.gov/content/sequence/LOCATE/locate.html)

**Data:** Coordinate listed according to the protein alignment of all 81 acutely infected subjects.

**Location:** Region in Envelope.

**Posterior Probability:** Sites identified in HyPhy with a posterior probability>0.5 are included in this table.

**P-value/q-value:** The p and q values from the Phylogenetic analysis for a specific mutational pattern being associated with an earlier or later Fiebig stage.

**Number of subjects with variation:** Out of the 81 subjects, the number that had any variation in this site.

**APOBEC3 in subject's mutations:** The number of subjects among those that vary in a given position that have a G to A change in the context of an APOBEC3 motif.

**Timing:** The selection results were obtained using all samples in Fiebig stages I–V.

For the phylogenetic method a change at the site was enriched in the range of Fiebig stages shown.

**Number of subjects with a mutational pattern:** This summarizes all individuals with changes found in this position in the data. *Italics* means a change was found more than once in at least one person with the pattern.

**Posterior probabilities:** The number of individuals that have a change from the most common amino acid to another. If there was more than 1 change in any of the individuals, it is noted in italics.

**Example 1:** Site 651: 2 N to S, 1 S to G means: Two people had N as the most common amino acid, but S was present. PRB931 had 17 N and 2 S, PRB956 had 25 N and 1 S. Because there was more than one S in one of them, it is in italics. One person, 700010077, in noted as S to G, and had 51 S and 1 G.

**Example 2:** Site 518: 2 M to I, 7 M to V means: Nine people had M as the most common amino acid, and each of the nine had a single variant among their sequences, and it was I in 2 of them, or V in nine of them. For example, subject 1012 had 42 sequences, with 41 M and 1 V.

**Phylogenetic method:** The within-subject change that was observed to be enriched either early or late Fiebig stages.

**Example:** Site 460: 2 Away from N means: In the subjects that had changes in this position, the ancestral state of the founding virus (the transmitted virus) was most likely to be N, and in both cases at least one change away from N was observed. Because it is italics, at least one of the two carried more than one mutation from N in this position.

**CTL testing:** If a variant was found multiple times in a patient or embedded in the context of additional proximal changes, peptides spanning the region were generated and T-cell assays were perform (see Table 3).

*: In Keele *et al.*, we noted patients bearing these changes might have been infected with more than one closely related form. Alternatively, early selection or maintenance of a very early mutational event might be giving rise to the pattern.

**: This site was difficult to align in a few patients due to a frameshifting insertion of an A in a string of As in the primary sequences, thus some of 7 noted E to K changes were actually due to a frameshifting indel.

***: In an additional subject there was an ambiguous base call in this position.

### Analysis of rapidly evolving sites

**Figure 1:**
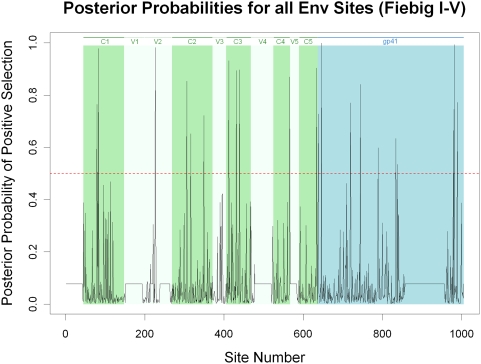
Posterior probabilities of belonging to the selection class (ω>1) for all sites in gp160. The dashed line indicates the 0.5 posterior probability, which we used as a threshold to assign sites to the selection site class. Flat parts of the graph correspond to sequence regions that were masked either because they were poorly aligned or coding in more than one frame.

**Figure 2:**
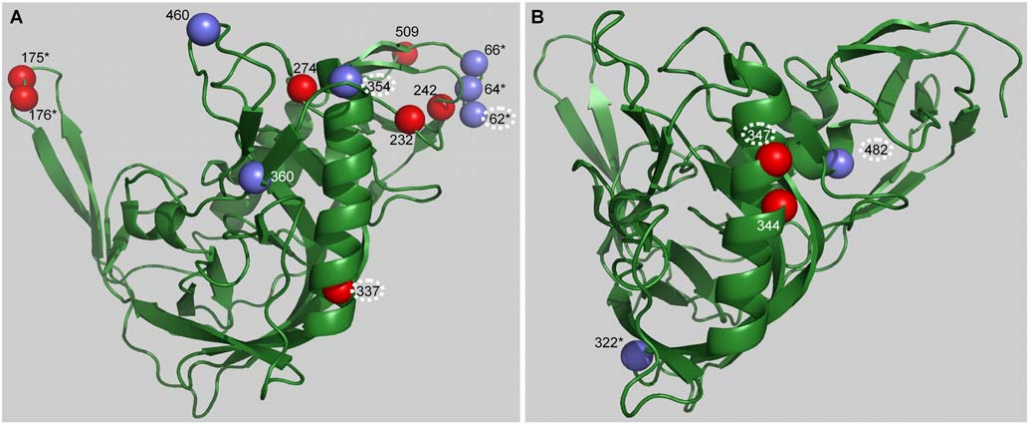
Three-dimensional structure context of the selected sites identified in gp120. (A) 13 sites from the dataset excluding sequences with evidence of APOBEC-mediated hypermutation, and (B) 4 additional sites identified analyzing the complete dataset. The sites depicted in blue represent those sites that are embedded in a known or potential CTL epitope and circled site numbers are potentially affected by APOBEC hypermutation. Sites marked with an asterisk occur in a region for which there is no available structure, therefore the positions are shown in proximity to their actual locations.

We found 24 sites in *env* with posterior probability >0.5 of belonging to the positive selection site class based on the 66 subjects discussed above (Table 2). Thirteen of these occurred in gp120 and eleven in gp41 (Figure 1). The three-dimensional structure context of the selected sites in gp120 is shown in Figure 2A. There is a cluster of sites in the C3 region of gp120 (Table 2) that are in the positive selection class. In general, sites evolving under positive selection appear to be at least partially solvent exposed (Figure 2A). Six additional sites were identified by including all 81 subjects in the analysis (Table 2, and Figure 2B); 4 were in gp120. For all of the sites identified we considered the sequence alignment carefully to assess the possibility that the evidence of positive selection could be resulting, for example, from APOBEC-mediated mutations or alignment errors associated with indel hotspots (Table S1, provides a detailed list of the observed mutations and their sequence context). For one site (at position 509 on the HXB2 sequence), alignment error introduced by an indel hotspot provides a better explanation of the data than selection. This site occurs at the beginning of a long sequence of Adenines that could serve as an indel hotspot [23] and mutations at this site could, in several cases, be more parsimoniously explained as indels. For 7 of the 24 identified sites (indicated in Table 2) some or all of the mutations involve G→A in the APOBEC3G or APOBEC3F hypermutation motif (the motif is GRD, where R is the IUPAC code for G or A, and D for G, A, or T). A baseline of 12.3% of the bases in the patients' transmitted sequences corresponded to a G embedded in an APOBEC3 motif, while 26.8% (25/93) of the distinct mutations found in the 24 sites identified as evolving under positive selection involved a G in an APOBEC3 motif. When all 81 patients were included, 12.3% of the bases in transmitted sequences were G's in an APOBEC3 motif, while 28.6% (32/112) of the observed changes in selected sites involved a G in an APOBEC3 motif. In contrast, G's not embedded in an APOBEC motif were not enriched among sites under positive selection relative to the transmitted virus. Indeed, the proportion of G's embedded in an APOBEC3 motif was significantly higher for the selected sites than for the remainder of the virus in both the 66 patient subset (Fisher's exact test p = 0.004), and the full 81 patient (p = 0.0002) data set. This suggests that some of the sites are identified as belonging to the positive selection class because of the elevated APOBEC3 mediated mutation. Because the waiting time for G→A mutations in the APOBEC3 context may be shorter than for other mutations, it is possible that selection acting on these mutations may be a hallmark of early immune escape.

**Figure 3:**
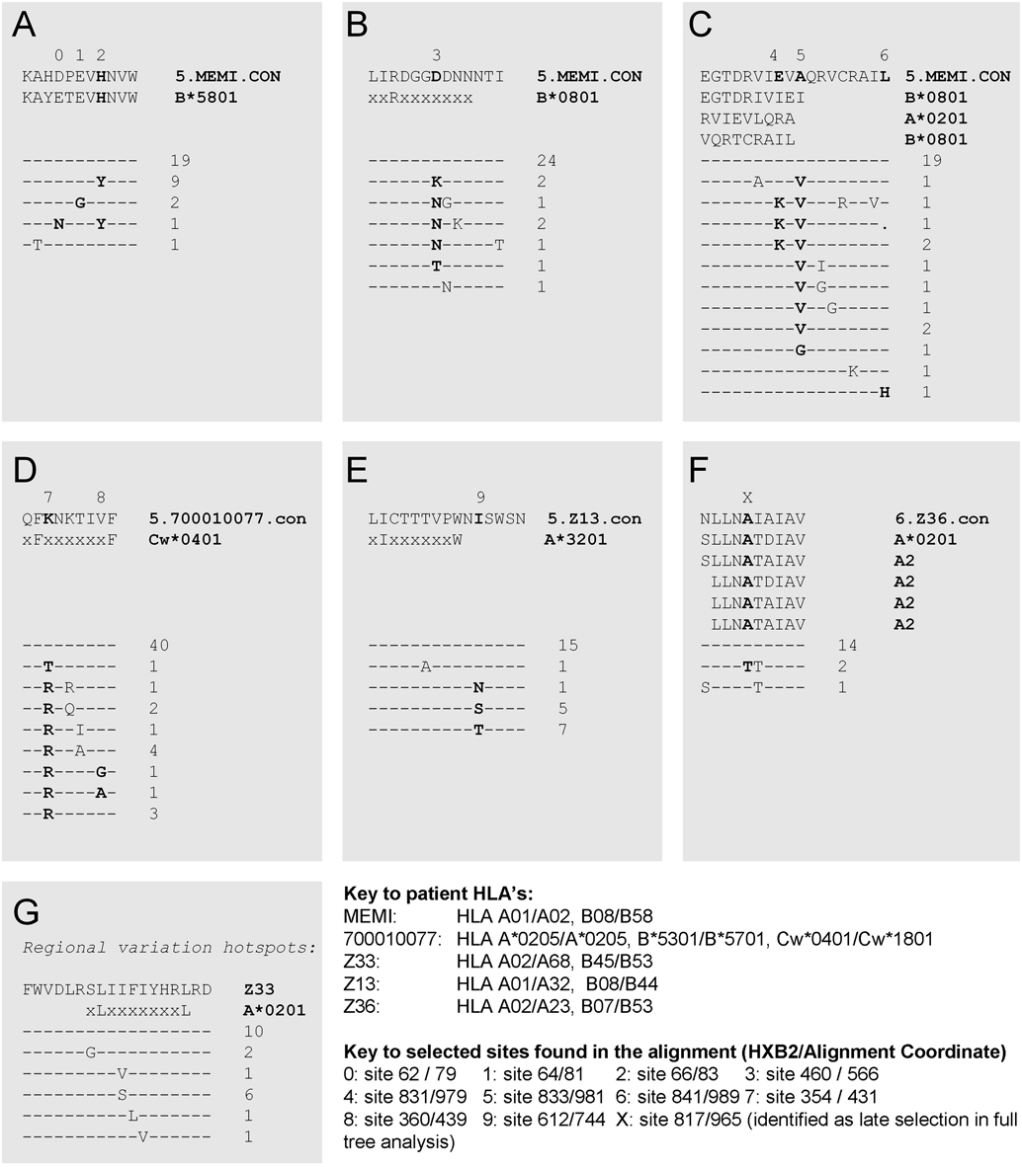
Selected sites that are embedded in potential CTL epitopes. The patient consensus sequence is at the top of each alignment, with Fiebig stage, patient ID, and CON for consensus indicated. The proposed epitope is shown beneath the patient consensus, followed by the HLA. Previously reported epitopes are provided in full, predicted epitopes are written with uppercase letters representing the anchor motif embedded in a string of x's. (A–E) are based on i) evidence for selection from the model implemented in HyPhy, ii) evidence of selection from locally concentrated regional evidence for selection relative to extremely homogeneous early infection alignments, and iii) HLA appropriate known epitopes or anchor motifs found using the Los Alamos HIV immunology database epitope location finder, ELF. (F) This potential escape mutation was found by the phylogenetic test for changes enriched in later Fiebig stages, and was also supported by (ii) and (iii) above (Table 2). Mutations in (A–C) also were favored in later Fiebig stages. (G) This set of mutations includes the only other sites in the entire data set of 81 subjects where mutations tended to cluster over a small region in one person. No site in this region had statistical support for positive selection, but this cluster of mutations was embedded in a potential epitope.

There were 10 positively selected sites that were located in 5 potential CTL epitopes. An additional site (position 965 in the alignment) that was detected as an enriched change in late infection (Figure 3F, also see below) was embedded in a 6^th^ potential epitope. These selected sites were investigated as potential CTL epitopes because each was embedded in a localized region with multiple mutations in one of the study subjects (Figure 3), and some of the variants were repeated multiple times within the subject (Figure 3), two features providing corroborative evidence for ongoing positive selection for immune escape. In each of these six cases, the cross-sectional sample appeared to capture a moment of transition in the viral population, with the virus exploring different escape options. In each of these cases, patient HLA data were available, and the clusters of mutations were found to occur either within a known CTL epitope from the literature, or within anchor motifs appropriate for the HLA type of the infected individual (all potential epitopes are shown in Figure 3 and summarized in Table 3, identified using the tool ELF http://www.hiv.lanl.gov/content/sequence/ELF/epitope_analyzer.html). As this was a cross-sectional study, the sequences from which we infer selective pressure are from the moment of transition, thus only direct experiments can determine which of the variants represent the susceptible form.

**Table 3:** CTL results indicating the six regions tested as well as the specific epitope sequences.

Alignment coordinate *APOBEC3*	Patient (Fiebig stage)	Sequences Tested (Database Analog)	Epitope Annotation	EliSpot SFU/10^6^ PBMCs	Cultured Elispot SFU/10^6^ PBMCs	ICS % Total CD8+ memory T-cells	Summary
***79***, 81, 83	MEMI (V)	KAHDPEVHNVW (B*5801)	Putative epitope	487	15144	N/A	Positive
		KAHDPEV**Y**NVW	Common variant - escape?	222	10227	N/A	Diminished
		ASDAKAHDPEVHNVWATH	18mer bound selected area	133	1655	N/A	Positive
		ASDAKAHDPEV**Y**NVWATH	18mer common transmitted variant	Negative	2086	N/A	Diminished
***431***, 439	700010077 (V)	QFRNKTIVF (Cw*0401)	Putative epitope	477	N/A	N/A	Positive
		REQFRNKTIVFNHSSGGD	18mer bound selected area	107	N/A	0.71 (32 d)	Positive
		REQF**K**NKTIVFNHSSGGD	18mer common transmitted variant	Negative	N/A	N/A	Negative
		LSHVVDKLREQF**K**NKTIV	18mer common transmitted variant	Negative	N/A	N/A	Negative
***979***, 981, 989	MEMI (V)	RVIEVAQRV (A*0201)	Putative epitope	Negative	9930	N/A	Positive
		RVI**K**V**V**QRV	Common variant - escape?	Negative	2468	N/A	Diminished
		EGTDRVIEVAQRVCRAIL	18mer bound selected area	Negative	Negative	N/A	Negative
		EGTDRVI**K**V**V**QRVCRAIL	18mer common transmitted variant	Negative	Negative	N/A	Negative
		EGTDRVIEVA (B*0801)	Putative epitope	Negative	Negative	N/A	Negative
		EGTDRVI**K**V**V**	Common variant - escape?	Negative	Negative	N/A	Negative
		AQRVCRAIL (B*0801)	Putative epitope	Negative	Negative	N/A	Negative
		**V**QRVCRAIL	Common variant - escape?	Negative	Negative	N/A	Negative
744	Z13 (V)	LICTTTVPW (A*3201)	Putative epitope	N/A	N/A	Negative	Negative
		GKLICTTTVPWNISWSNK	18mer bound selected area	572	N/A	N/A	Positive
		GKLICTTTVPWN**T**SWSNK	18mer common transmitted variant	N/A	N/A	Negative	Negative
965	Z36 (V)	LLNAIAIAV (A*02)	Putative epitope	N/A	N/A	Negative	Negative
		KNSAVNLLNAIAIAVAEG	18mer bound selected area	N/A	N/A	Negative	Negative
		KNSAVNLLN**TT**AIAVAEG	18mer common transmitted variant	N/A	N/A	Negative	Negative
566	MEMI (V)	LIRDGGDDNN (B*0801)	Putative epitope	Negative	Negative	N/A	Negative
		LIRDGGD**N**N**K**	Common variant - escape?	Negative	Negative	N/A	Negative
		LIRDGGDDNNNTIEIFRP	18mer bound selected area	Negative	Negative	N/A	Negative
		LIRDGGD**N**N**K**NTIEIFRP	18mer common transmitted variant	Negative	Negative	N/A	Negative

Sites that occur in the APOBEC3 context are shown in italics.

To determine if there indeed was a T-cell response to these putative epitopes, 18 amino acid long peptides representing the most common and second most common form of the regional variants encompassing the variable sites, as well as the predicted epitopes shown in Figure 3, were synthesized and tested using either ELISpot or ICS (see Methods). For example, three sites (79, 81 and 83 in the alignment), showing evidence of adaptive evolution (Table 2) were mutated in patient MEMI. These sites were embedded within a previously described B*5801 epitope, and MEMI carried HLA B*5801 (Figure 3A). Upon testing, MEMI in fact did make a T-cell response to the most common form of the B*5801 epitope (KAHDPEV**H**NVW) and a diminished response to the variant KAHDPEV***Y***NVW (Table 3). We were able to detect a T-cell response to 4 of the 6 proposed epitopes we tested (Table 3). The lack of the response to the other 2 proposed epitopes could imply an alternative cause for the selection at this sites (e.g. antibody escape or reversion). However, it is also possible that all of the observed forms of the peptide are escape variants, and that the susceptible form has decreased in frequency to the extent that it does not appear in the sample and was therefore not tested. Further possible explanations include a transient T-cell response driving the escape that was already beginning to subside due to immune escape and reduced stimulation, or limited sample viability (this was a retrospective study, and the condition of several of the samples was not ideal).

Interestingly, 3 of the 4 validated T-cell escape forms included a G→A mutation embedded in APOBEC3G or 3F motif, a further indication that an APOBEC-mediated enhanced mutation rate may facilitate early CTL immune escape in some cases (Table 3). Clusters of mutations that co-occur in a patient may represent an alternate way in which immune escape can be inferred and is consistent with previously observed patterns of escape from an immune response early in infection [24]. Again, patient MEMI provides an example, with at least three independent nonsynonymous substitutions at site 566, which was embedded in a proposed but not validated epitope for one of the HLA alleles of this patient (B*0801). Collectively the results suggest that there is strong evidence for selection preferentially occurring within CTL epitope regions relatively early in infection – all potential CTL escape variants were found among Fiebig stage V cases in this study (Table 3).

The 12 other sites that were identified cannot be explained by APOBEC mediation and do not occur within mutation clusters and were therefore not investigated as possible CTL epitopes. Two sites (412 and 838 in the alignment) are identified largely because of mutations in patients Z31 and TT31P, where infection may have resulted from multiple closely related strains of the virus, rather than a single viral strain [15]. For all but two of the remaining sites, the observed mutation occurred on a single terminal branch of the within-patient phylogenetic tree and mutations were consequently observed only once per patient. These sites were identified as under positive selection because the site was mutated in multiple patients. Thus, unlike the substitution patterns in the putative CTL epitopes described above, there was no evidence at this stage of infection that these mutations were spreading in the intra-patient viral populations. As a result, these sites may be mutational hotspots, albeit hotspots that cannot be explained by known mechanisms of hypermutation in HIV-1. Note that two patients may share a mutation at a given site by chance with a probability of 0.12 (calculation described in the Methods section), but the probability falls off rapidly as more patients are observed to share a mutation at the same site. The probability of three patients sharing a mutation at the same site is 0.03, and for four patients this drops to 0.004. Even then, seeing, for example, seven changes within a codon (four at one position and three at another) *once* in a sequence of length 2568 (the median sequence length) is quite likely (p = 0.45). Using this approach, only at one site in the alignment (646, which is codon 518 in the HXB2 strain) do we find a significantly greater number of patients sharing a mutation than can be explained by chance (nine patients have a mutation at this codon, shared as seven at one codon position and two at another; p = 0.003).

**Figure 4:**
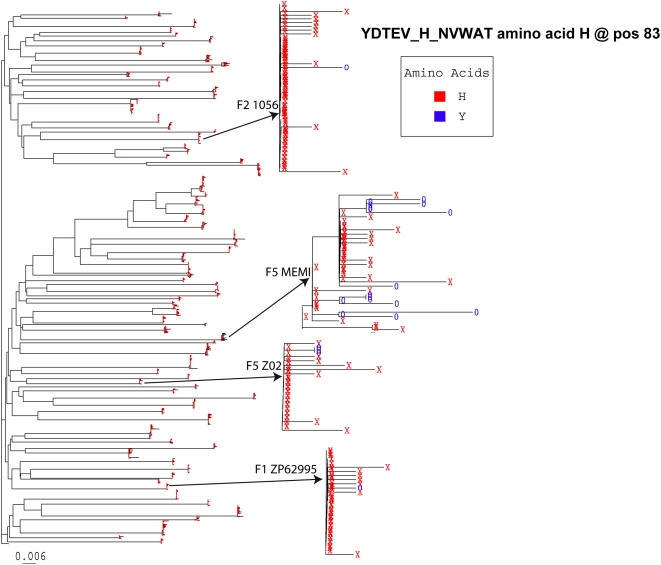
Maximum likelihood tree including all sequences from all 81 homogeneous patients illustrating the pattern of change in one site. The tree is based on all nucleotide sequences and all positions in Env, and illustrates the mutational patterns in the context of the full tree at amino acid position 83. Position 83 is highly conserved, and is His in most sequences in most subjects (indicated by the red Xs). In the 4 subjects that are featured, it changes from His to Tyr in at least some of the sequences in each subject, indicated by the blue 0 s. Although the trees were generated for each patient separately for the HyPhy analysis, this kind of repeated mutation pattern in a single site is an illustration of the kind of change captured in the dN/dS ratio of the HyPhy analysis (Table 2). This site, 83, was also identified as interesting in the full tree analysis as it tended to change from H to Y in patients at later Fiebig stages (Table 2).

We used a separate phylogenetic method to identify sites that appear to have a different rate of mutation in the initial, compared to later clinical stages of early infection (Table 2). This analysis was based on the maximum likelihood tree including all the sequences from the 81 homogeneous patients (an example of the substitution pattern in one position is illustrated in Figure 4). Changes were identified relative to the maximum likelihood estimate of the transmitted virus in each individual. Mutations that tended to occur more often in either later or earlier Fiebig stages were indentified. Eight sites were mutated more frequently in later Fiebig stages (III–VI), thus these were potentially selected by immune pressure, while only one change recurred preferentially in Fiebig I–II (in patient TT31P at position 454 in the alignment). In this case, the subject had 67 sequences available, 49 carried an Alanine in this position and 18 a Valine. Alanine, which is found in the putative infecting virus, is very rare in the population at this position, so the mutation is a candidate for early reversion to a more fit form. In patient TT31P the mutation from Alanine to Valine appears to have occurred more than once in independent lineages, suggesting strong selection in this patient at this site in early infection; however, this could also be explained by transmission of two variants together with recombination prior to, or soon after, transmission. The 8 sites highlighted using this method included 4 that were among the sites identified by the HyPhy method as well as one additional site that may be the results of CTL escape as discussed above (Figure 3F).

We assessed by visual inspection whether there was evidence of directional evolution in any of the 24 rapidly evolving sites identified above (i.e. a consistent trend towards replacement of one amino acid by another amino acid across multiple patients). Position 646 (518 in HXB2), which occurs in the fusion peptide of gp41, showed consistent replacement of Methionine with either Valine or Isoleucine (the observed substitutions relative to the within patient consensus are summarized for each selected site in Table S1). This trend is remarkable when the data from all patients is considered. Fewer than half (38 of 81) of the patients had Methionine at this position in the inferred infecting viruses, yet all nine of the patients with a nonsynonymous mutation at this site had Methionine in the patient consensus sequence. The probability of this occurring by chance is 0.001. Such strong directional selection in early infection suggests the possibility of reversion of amino acids associated with a selective transmission bottleneck, but could also be explained by reversion of an escape mutation associated with an immune response in the transmitting virus. The apparent directionality could also be explained by a sequence-specific mutational hotspot if the ATG codon, encoding Methionine, results in a sequence motif that promotes mutation. This is suggested by the fact that the mutation from M to V or I is found in just a single sequence in each of the patients in which it occurs and thus does not appear to show evidence of spreading in the intra-patient viral population. However, none of the mutations at this site are consistent with APOBEC3G/F mutation, and thus rapid evolution at this site cannot be explained by known mechanisms of hypermutation.

Since the number of potential N-linked glycosylation sites (PNGSs) in *env* may affect the fitness of the virus by rendering it more or less sensitive to neutralizing antibodies, we tested for enrichment of mutations within PNGSs. The observed number of mutations within PNGSs (NX[S|T]X where X is any amino acid other than Proline) was not significantly different than the expectation under a model of random mutation. We, therefore, found no evidence of a tendency for mutations to disrupt PNGSs in early infection. (p = 0.736; Fisher's exact test). We applied a similar approach to determine whether there was an overall enrichment of mutations within epitope-dense regions. In this case, we compared the number of mutations that occur within epitope rich regions (coordinates based on the best defined CTL/CD8+ epitope summary [25]) to the total number of mutations found outside the epitope regions. There was no evidence of enrichment of mutations occurring within a CTL epitope-dense region as opposed to less dense regions (p = 0.933, Fisher's exact test). However, because all mutations were considered in this case, the lack of such evidence may result from the high level of noise in the data (i.e. the fact that the majority of the observed mutations are likely to be neutral or under purifying selection, rather than positively selected). When we consider only the 24 rapidly evolving sites, we find a weak trend (p = 0.27) towards enrichment within epitope rich regions (odds ratio: 1.6).

### Analysis of insertions and deletions

**Figure 5:**
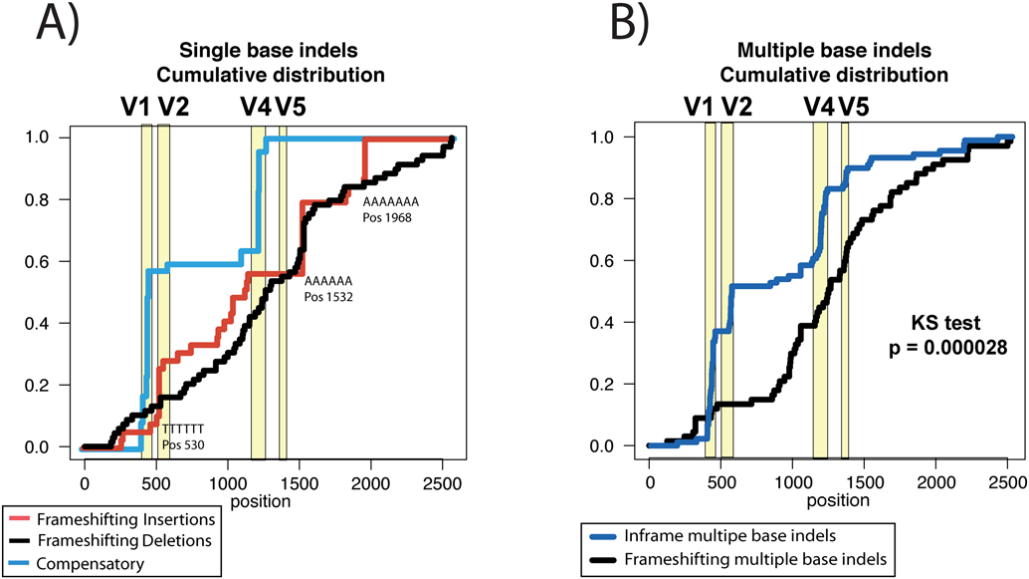
Cumulative distribution of insertions and deletions in acutely infected patients. (A) The frameshifting single base indels observed in early infection were distributed throughout the Env gene, with a few concentrated regions localized in particular strings of like bases (red and black lines in the figure). As these substitutions are lethal, they must have arisen in the newly infected individual. In contrast, most of the single base indels in the 21 heterogeneous infection cases were compensatory, associated with nearby mutations that resolved the reading frame, and most of the compensated indels are localized in the hypervariable domains. (B) Similarly, multiple base frameshifting indels are distributed throughout Env, while in-frame indels are concentrated in the hypervariable regions.

For the homogeneous samples, the within-subject consensus is a good approximation of the transmitted virus [15], and therefore the direction of an insertion or deletion can be clearly ascertained. Unique indels in these cases are one round of replication from a viable virus, but they are most often frameshifting, and clearly lethal, indicating they must have arisen in the newly infected host and often are an evolutionary dead end. Most frameshifting indels occurred only once within a patient, with the exception of single base insertions and deletions that can occur in runs of like-bases, most often in strings of A's, although one hotspot is in a string of 6 T's. It is difficult to resolve the relative frequency of such patterns that result from in vivo mutations versus experimental artifacts introduced during cDNA synthesis. Among the 81 homogeneous infections, there were 31 observed single base insertions; 11 are unique, and 20 were recurrent and distributed among a few hotspots (Figure 5A). There were 53 single base deletions (1.7 fold more common than insertions), 44 were unique, and 13 were found in hotspots at runs of like-bases. All these indels were lethal and frameshifting. In contrast, within the 21 heterogeneous patients that carry indels that could have arisen in the donor, there were 44 single base insertions, but 30 were compensatory and resolved a nearby frameshift to keep the reading frame intact. There were 32 single base deletions, and 17 were compensatory mutations resolving a nearby frameshift. Hence, the single base indels that were compensatory were found exclusively among the heterogeneous cases, and thus were likely to represent viable substitutions carried in through transmitted viruses. Unlike the lethal single base indels, which were found distributed throughout *env* but with a bias towards strings of like bases, the compensatory single base indels were not proximal to like bases, were found exclusively in heterogeneous infections, and were all concentrated in the hypervariable V1, V2 and V4 loops (Figure 5A). This suggests that single base indel mutations occur throughout *env*, but are concentrated by selection in the hypervariable domains.

Multiple base indels showed even more remarkable contrasts between homogeneous and heterogeneous infections. In the homogeneous subjects, only 10 multiple base insertions were found and all were unique events. Four, i.e. about a third, as would be expected by chance, were in-frame and none compensated for other frameshifts. Nine of the 10 were direct repeats of a nearby stretch, although not always adjacent. In the following two examples the region in bold is the region from the transmitted strain that is repeated in the brackets:

TACTTGGAATA *GTACTTGGAATA* [ *GTACTTGGAATA* ] ATACTGAAGGGTC
GGTAAC *GGT* [ *GGT* ] AACGAGACC

In contrast, in heterogeneous infections there were 198 insertions, resulting from 49 unique patient/insertion combinations (i.e. many of the insertions were observed in multiple sequences from the same patient). Of the 49 unique insertions, 43 were in-frame, and the other six each had a nearby compensatory mutation. There were many more multiple base deletions among the homogeneous patients; 115 were found, again all unique, with 33 very large deletions (median 217 base pairs; range 40–601 base pairs) that were presumably lethal. Of the 82 smaller deletions, 27 were in-frame (33%) and 55 were frameshifting. In contrast, in the heterogeneous infections there were 289 deletions resulting from 82 unique patient/deletion combinations. Among these unique deletions 56 were in-frame and 53 of these were in V1, V2 and V4. Another 7 deletions were compensated by nearby indels. The in-frame and out-of-frame multiple base indels were distributed very differently across the genome (Figure 5B; Kolmogorov-Smirnov p = 0.00003). Out-of-frame indels were distributed throughout Env but the short, presumably viable, in-frame ones were concentrated in the hypervariable loops. This provides strong evidence that indels do not occur as hotspots with a preference for the hypervariable domains, but instead are preferentially maintained in the hypervariable regions, presumably because of selective constraints that prevent length variation in other regions and, perhaps because length variation is selectively advantageous in these regions.

## Discussion

The availability of data from a large number of individuals in acute and early infection that harbor a homogeneous set of viruses leads to an opportunity to study the pattern of diversification of HIV-1 upon transmission to a new host. Because 78 of these patients are believed to have been productively infected by single viruses (three others are likely to have been infected by two closely related viruses) the observed diversity can be inferred to have arisen in the newly infected patient, and not in the individual from whom the infection was acquired [15]. The size of the sample and the quality of the sequence data provide the first opportunity to assess whether there are consistent patterns of evolution that tend to recur across multiple patients in early infection. Knowledge of the infecting virus not only enables us to discriminate the direction of the mutations but also allows us to distinguish between insertions and deletions, which are likely to be a significant aspect of HIV diversification in early infection. Differences in the lengths of *env* variable loops between viruses in acute and chronic infection have previously been reported in some subtypes [19],[20],[26] and this may play a role in increased neutralization sensitivity of the virus in acute infection. Such differences, if they exist, could result from selective transmission of viruses with shorter variable loops, or alternatively from selective events during acute infection.

We used HyPhy [18] to assess the selective pressure acting on viruses in early infection. In our model we constrained ω, the ratio of nonsynonymous to synonymous substitution rates, to be the same at a given site in the codon alignment across all patients. This allowed us to identify sites that diversify rapidly on average. The method we developed is sensitive to multiple independent mutations at the same site in a given patient, or to the mutations at the same site in multiple patients, or a combination of the two. Because the sequence datasets from acute infection are very homogeneous and generally well described by a star-like phylogeny, it was rare to find multiple independent mutations within the same patient and consequently we primarily identified sites that tended to diversify rapidly in many patients. Many of the sites we identified coincide with experimentally confirmed CTL responses, indicating the importance of CTL escape early in infection. However, the sites that we detected are likely to represent only a fraction of the mutations that have been driven to detectable frequency as a consequence of immune escape. For example, because our method relies on shared patterns of mutation across multiple patients we are unlikely to have power to detect escape from immune responses mediated by rare HLA alleles. Additionally, since parts of *env*, particularly in the variable loops, were masked from the analysis due to ambiguous sequence alignment, sites that confer resistance to neutralizing antibody escape may have been overlooked. Although our results underscore the importance of CTL escape in early infection they do not provide a basis to discount the potential contribution of escape from antibody responses to the evolution of *env* early in HIV-1 infection.

We used statistical model comparison to make the case that there is evidence of adaptive evolution in HIV-1. To do this we compared the fit to the data of a model in which no sites in the alignment were allowed to evolve with ω>1 to the fit of a model in which a subset of sites were allowed to have ω>1. The latter model had a significantly better fit to the data for the sequences from Fiebig stages I–III and Fiebig stages I–V, suggesting that the virus evolves adaptively in these clinical stages. However, there are two important issues to consider here. First, the fact that the positive selection model did not have a significantly better fit to the data in Fiebig stages I–II does not prove that viral diversification is not shaped by positive selection at this stage of infection. Sequences from stages I–II were highly homogeneous, thus a very small number of mutations from the infecting virus were observed in each patient, severely limiting the power to detect evidence of selection. Secondly, a more general issue is that models of codon evolution used to detect positive selection rely upon several assumptions about the way the sequences are evolving and deviations from these assumptions could lead to false positive results [27]. APOBEC induced mutations give rise to hotspots of amino acid change which have a mutational rather than a selective cause. Although we attempted to control for the effects of hypermutation by removing sequences and patients with evidence of an elevated rate of mutation in the APOBEC3 sequence context (Figure S1), the enrichment of mutations in this context among sites placed in the positive selection site class suggests that hypermutation may have had an effect on the model comparison results.

The phylogenetic codon model we applied using HyPhy does not account for recombination, which is known to cause false positive inference of selection [28],[29]. With the highly homogeneous intra-patient datasets analyzed here, recombination is not likely to have a large effect, because the sequences are, in most cases, well described by a star phylogeny, with little internal structure in the tree. However, in the case of the later Fiebig stage patients there is some tree structure and recombination in these patients may have the potential to cause false inference of positive selection. We therefore tested all the homogeneous intra-patient sequence data sets for evidence of recombination using GARD [30]. We found evidence of a single recombination breakpoint in the sequence alignments from each of two patients (Z31 and MEMI). In both cases the breakpoint is located near the end of the sequence alignment (just 3% and 10% of the alignment lies to the right of the breakpoint for MEMI and Z31, respectively) and therefore a single tree topology applies for most of the alignment. Only in the case of MEMI do nucleotide mutations occur to the right of the recombination breakpoint and at inferred adaptively evolving sites (979, 981 and 989). Since these mutations are also clustered and two of the three are confirmed CTL escape mutations (Table 3), it appears unlikely that these sites are false positives.

We tested for evidence of directional change in *env* in early infection since this could impact our understanding of the nature of the transmitted virus. It is clear from several studies [15],[20],[26],[31] that there is a severe viral population bottleneck during HIV transmission, with newly transmitted infections resulting frequently from transmission or outgrowth of a single virion. If this extreme bottleneck is non-random and if viruses that are best adapted for transmission from one host to the next tend to be less well adapted for replication in the new host, then there may be selection for reversion of the characteristics associated with transmission in the newly infected host. If, further, these characteristics are shared across multiple transmitted viruses, then such reversions in early infection could potentially be observed through our approach. We found evidence for purifying selection, indicating the transmitted virus was a reasonably fit form prior to the initial immune response imposing new selection constraints, and we found relatively little support for characteristic patterns of reversion at the population level in this cross-sectional view.

Discounting sites that may have been affected by APOBEC3G/F hypermutation we found just a single site with significant evidence for directional change. At position 646, (518 in HXB2), which is in the fusion peptide of gp41, we found consistent mutations from Methionine to Valine or Isoleucine. However, at this site, there is just a single mutant sequence observable in each of the nine patients in which the mutation is found, and this seems inconsistent with a mutation that is coming under selection for increased frequency in multiple patients. Furthermore, we do not observe an increased frequency of Methionine in acute sequences compared with chronic sequences at this site (data not shown), as would be expected if the consistent mutations from Methionine to Isoleucine or Valine represented reversion associated with selective viral transmission. A single site was observed to undergo recurrent change in a single subject in Fiebig stage II, and as this is prior to the immune response it may be indicative of rapid reversion. Alternatively, the observation could be explained instead by transmission of two highly related forms, together with recombination either in the donor or soon after transmission.

Neutralizing antibody responses drive the evolution of viral escape and are therefore thought to play an important role in shaping the evolutionary changes observed in the envelope gene. Mutations, insertions and deletions, as well as the distribution of glycosylation sites along the envelope gene mediate the ability of the virus to escape from neutralizing antibodies although the relative contribution of each of these is unknown [32]. The role of N-linked glycosylation sites in the *env* gene in maintaining the survival of HIV-1 during the earliest stages of infection is of particular interest as the carbohydrate surface establishes the first means of contact between the host and the virus. A previous study [32] compared the rate of phenotypic escape of HIV-1 in recently infected individuals from neutralizing antibodies to various characteristics of the envelope gene and found that escape from neutralizing antibodies can occur regardless of the variation in glycosylation or rate of insertions and deletions in *env*. We did not find evidence of accelerated diversification at potential N-linked glycosylation sites in early infection, which suggests that if escape from neutralizing antibodies is among the major driving forces of HIV-1 evolution at this stage the mechanism is unlikely to be primarily through single base substitutions in glycosylation sites, at least in the portion of *env* that could be aligned with high confidence.

The majority of the rapidly evolving sites that recurred within individuals were clustered and appeared to be under selection for escape from cellular immune responses, and several of these mutations are G→A substitutions in APOBEC3 motifs. We also observed a highly significant enrichment overall for APOBEC3 motifs among the rapidly diversifying sites identified using HyPhy, even when stringent precautions were taken to exclude the 15 subjects that had enrichment of G→A mutations within the APOBEC3 motif. This suggests the possibility that APOBEC3 and imperfect Vif function can play an important biological role in the patterns of mutation found in early infection, and may contribute to determining the first immune escape mutations. Thus base mutation rate may be in some cases making a contribution to determining which immune escape events happen first, along with fitness [33] and possibly T-cell potency [34]. CTL immune responses have been shown to play a key role in controlling viral replication in acute infection [35]–[38] and, in animal models, positively selected CTL escape variants have been reported within weeks of infection [39]–[41]. Escape from CTL responses has been observed in humans both early and later in infection [42],[43] and escape from successive immune responses is an important feature of HIV disease progression.

In our dataset several of the rapidly evolving sites are associated with clusters of mutations that occur in close proximity to one another in the same patient (Figure 3). In some cases there are multiple escape mutations at the same site. Given the short times since infection in these patients and the otherwise highly homogeneous sequence alignments, the multiplicity of alternative CTL escape pathways that are already beginning to be explored by the virus in early infection is remarkable. Several studies have also shown that upon transmission some HIV-1 escape mutations can revert to the consensus in order to re-establish effective replication efficiency within the new host [2], [44]–[46]. These reversion mutations frequently occur in structurally conserved regions and often reside within defined CD8 epitopes that are no longer subject to the restrictions of the original host's HLAs [2],[44],[45],[47]. Envelope evolution towards the wild-type sequence state after transmission is thought to occur in the absence of specific CTL mediated immune pressure [34],[48]. Even when the newly infected host has the HLA allele associated with the response, reversion can occur followed by development of a CTL response targeting the wild-type epitope. Given that essentially all examples of positive selection that also exhibited recurrent and localized concentrated change in these 81 individuals occurred within one of 4 experimentally validated CTL epitopes, or in one of 2 putative epitopes based on the individuals HLA, in this cross-sectional study the earliest changes seemed to result more commonly from escape rather than from reversion. However only in cases where CTL escape was confirmed experimentally can we conclude with certainty that the mutations are associated with escape.

In this study we have also shown that inactivating indels are distributed evenly through *env*, with the exception of single base indels in stretches of like-bases, which may in part be attributable to experimental artifact. On the other hand, in-frame and compensated indels are highly concentrated in 4 hypervariable domains in the heterogeneous infections leading to extensive length variation in V1, V2, V4 and V5. Because this was exclusively found in heterogeneous infections, we assume the length variants most likely arose in the newly infected individual's partner, at a later phase of infection. This indicates that there is no molecular trigger that increases the background rate of indels in the hypervariable regions during reverse transcriptions, and that the concentration of length variation in the hypervariable domains is instead a consequence of selection. Also this data does not provide evidence for selection for length variation during acute infection, suggesting that this may occur during the chronic phase of HIV-1 infection.

This cross-sectional study has provided a glimpse into the nature of the diversification of HIV-1 following transmission in a population of 81 B clade infected individuals. As expected purifying selection acts on single base mutations at the majority of sites as well as on indels, but there is also evidence of a subset of sites that diversify more rapidly than expected under a model of neutral evolution. Our results suggest that APOBEC3 may play a role in shaping the dynamics of early T-cell immune escape. APOBEC3 motifs are highly enriched among the rapidly diversifying sites. This could be the result of an anomalously high mutation rate giving the appearance of selection pressure when there is none, or a higher mutation rate promoting a particular route of immune escape; the two are not mutually exclusive. Finally early T-cell escape appears to be much more common than reversion. We provide new evidence supporting the idea that concentrated regions of change within an individual during early infection [24] are frequently indicative of early escape from T-cell responses.

## Methods

*Envelope* sequences (n = 3476) from 102 individuals infected with HIV-1 subtype B, were generated using Single Genome Amplification (SGA), as part of a previous study [15]. Among these individuals, 81 were found to be likely to have been productively infected by just a single virion (or infected cell), or two closely related viruses, while 21 were multiply infected since the level of diversity seen in these cases was greater than could have accrued since the time of infection. The 21 heterogeneous cases were not included in the analysis of selection pressure, but were used for a comparison of indel patterns. Intra-patient alignments were generated through an iterative process, which included the alignment of all patient consensus sequences, manual editing, and re-alignment of each patients' sequence data to the associated consensus, as described in the supplementary data of Keele *et al.* [15]. Recombination analysis was carried out with GARD [30] and evidence of recombination was found in just two of the homogeneous patients. A further 14 from the heterogeneous group (analyzed previously [15]) showed evidence of recombination that was confirmed by either GARD [30] or Recco [49]. For our previous study, acutely infected patients were staged using clinical criteria described by Fiebig *et al.* [17], into six clinical stages from the earliest, Fiebig stage I, to the latest stage of early infection, Fiebig stage VI. All regions of the alignment that are translated in more than one frame, as well as all regions that were ambiguously aligned, were masked for the analysis of selection pressure. In addition, for model-based as well as the non-parametric inference of selection, described later, individual sequences that showed statistically significant evidence of APOBEC-mediated hypermutation using Hypermut 2.0 (http://www.hiv.lanl.gov/content/sequence/HYPERMUT/hypermut.html) were discarded. Among the patients, 15 had a generalized increase in the rate of G→A mutations in the context of the sequence motif associated with APOBEC hypermutation. In these cases there was an excess of G→A mutations in the APOBEC sequence context across all sequences from the patients and not concentrated in individual severely hypermutated sequences [15]. Since in these cases APOBEC mediated mutations may be occurring at an elevated rate, and this context-specific rate would deviate from the evolutionary model, we carried out a separate analysis of selection excluding these 15 patients. On the other hand, recurrent G→A mutations in the context of APOBEC substitution motifs are enriched overall in these data, suggesting APOBEC may have a role in HIV-1 evolution in acute infection, thus we analyzed in parallel the full set of 81 subjects. The alignments used for these analyses were from our previous study [15] and are available at http://www.hiv.lanl.gov/content/sequence/hiv/user_alignments/keele.

### Model-based inference of selection in early infection

We used HyPhy [18] to assess the evidence for a subset of codon sites in the *env* gene evolving under the influence of positive Darwinian selection [50] in early infection. Essentially, this method works by fitting standard codon models [51] of sequence evolution to multiple sequence alignments from all of the patients. The critical parameter of the codon models is ω, the ratio of nonsynonymous to synonymous substitution rates. If synonymous substitutions are assumed to be generally neutral, then sites for which ω is significantly greater than one are inferred to be evolving under positive Darwinian selection [52],[53], although deviation from the evolutionary model as a result of enhanced mutation rates at specific sites, for example, those embedded in APOBEC signature motifs, can also contribute in some cases to an elevated ω (see Discussion). We used the random effects likelihood approach [54] and a model of site-to-site variation in ω equivalent to Model M2a from Wong *et al.* [55]. The selection parameter, ω, was modeled using a discrete distribution with three classes: a purifying selection class for which ω, constrained to be less than one, is estimated from the data; a neutral class for which ω = 1 and a positive selection class for which ω is estimate from the data and constrained to be greater than one. The fit of this model is compared using the likelihood ratio test to a model, equivalent to M1a from Wong *et al.* [55], in which the proportion of sites in the positive selection category is fixed at zero. Both of these are mixture models. In the standard implementation of these models the likelihood of the data is given by
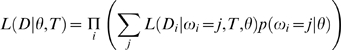
where the product is taken over sites of the alignment, the sum is over the discrete ω classes and *D_i_*, 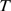 and *θ* are the data at site *i* in the alignment, the phylogenetic tree and the parameters of the model, respectively. We extend this to data from multiple patients by writing the likelihood as
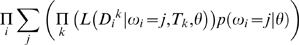
where *D_i_^k^* and 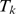 are the data and tree from patient *k*, respectively. In this formulation ω has the same value at a given site across all within-patient sequence alignments. Following likelihood parameter estimation, posterior probabilities of belonging to each of the site classes of ω were estimated for each site using the naïve empirical Bayes method [56]. For all of the models, mutation rates were estimated separately for each pair of nucleotides, thus assuming a general time-reversible model of nucleotide substitution and the Goldman and Yang method [57] was used to account for biased codon usage.

### Maximum likelihood phylogeny-based comparison of mutations in the earliest versus later clinical stages

To identify sites that showed different patterns of amino acid change comparing samples from earlier versus later Fiebig stages, we included the full dataset in a maximum likelihood tree based on an alignment of all sequences from the 81 homogeneous patients. We excluded extremely hypermutated sequences, but left in the 15 patients that showed overall enrichment for G→A mutations in an APOBEC context. Phylogenetic trees used for this analysis were inferred using PhyML [58], with a GTR nucleotide substitution model and a gamma distribution for site-to-site rate variation [59]. We iteratively improved the model and the tree, using a GTR model for base change with a likelihood estimate for the rate at each site, which does not assume a particular distribution [60]. We then identified amino acid changes from the transmitted virus that were preferentially associated with either early or late Fiebig stages using a modification of the contingency table approach as described previously [59]. Maximum likelihood estimates of the most recent common ancestor (MRCA), or founder virus, of each patient were generated and these were used as a baseline to determine the number of within patient changes between the MRCA and the tip at each position in each patient. Substitutions are counted by taking each taxon and following it back into the tree until an amino acid change is observed, or until the ancestor hits the MRCA, whichever comes first. For any taxon, there is an associated node in the tree for the substitution, and tabulation is done in terms of unique identities of these substitution nodes, so each change on an interior branch is counted only once. The total number of changes was tallied across all patients, and Fisher's exact test was used to compare the number of changes away from or towards each amino acid observed in patients at later Fiebig stages against the number of the same changes in patients at earlier Fiebig stages. All amino acid comparisons were made at each site in the alignment, and various comparisons between Fiebig stages were considered: Fiebig I versus II–VI, I–II versus III–VI, I–III versus IV–VI, I–IV versus V–VI, and I–V versus VI. The impact of multiple tests was addressed by requiring a false discovery rate, or q-value, of <0.2 [61].

### Probability of finding repeated changes in a particular position over the entire data set by chance

To calculate the probability of finding mutations repeatedly in multiple patients at a given site in the absence of selection, we assumed an exponential growth model with a large reproductive ratio. In such a case, a small number of sequences sampled from a patient are overwhelmingly likely to have a star phylogeny. Since in a star phylogeny every mutation arises independently, the probability of seeing a position mutated in at least one out of *s* sequences sampled *g* generations from a common ancestor is 
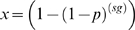
, where 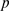 is the rate of mutation per generation per position. The generation time (2 days [62]) and mutation rate (2.16×10^−5^ [63]), were the same as those used in Keele *et al.* [15]. The probability of seeing this in at least *n* out of 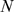 patients is 
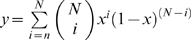
. The probability of seeing one such position in a sequence of length *L* is 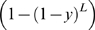. When the resulting probability is small, it can be approximated by 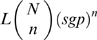. If the mutations are clustered in two sites in a codon, instead of at one site, 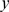 needs to be replaced by 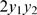 where 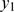 and 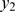 are the respective probabilities for the two sites and the approximate resulting probability is 
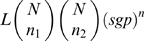
, when the *n* changes are split as *n_1_* and *n_2_* at the two sites. In the calculations presented here, we used computer code to generalize these results to the case when each patient had a different number of sequences and generations since the last common ancestor.

### Indel analysis

For this analysis, all insertions and deletions (indels) in each subject relative to the subject consensus were systematically tracked, and information regarding their location, length, and the precise inserted or deleted fragments, and surrounded bases were retained and analyzed. The data were divided into 4 sets, single base insertions and deletions, and multiple base insertions and deletions, and each was considered separately. For the sequences derived from the 81 homogeneous early infections, the direction of the indel could be ascertained relative to the consensus, which was presumed to be the transmitted strain, and observed indels were assumed to be random events within the study subject. This assumption was supported by the high frequency of frameshifting inactivating substitutions among indels in the homogeneous infections. In contrast, the 21 heterogeneous infections had an excess of indels that were in-frame and thus more likely to be viable, and so probably arose in the donor and were transmitted independently to the recipient.

### ELISpot assays

#### Ex-vivo IFN-γ ELISpot assays

18mer and 9mer peptides were synthesized (MRC Human Immunology Unit, WIMM, Oxford UK) to match sequences from patients MEMI, Z13, Z33 and Z36. Cryopreserved PBMC samples were thawed and rested for 2 hours before being placed in the ELISpot plates at 1×105 cells/well. Peptides in the peptide plates were mixed with 1∶1 with PBMC in the ELISpot plate to a final concentration of 2 µg/ml and incubated for 20 hours at 37°C, 5% CO_2_. PBMCs and peptides were tested in R-10 medium (10% fetal bovine serum, 86% RPMI 1640, 2 mM L-glutamine, 1× penicillin-streptomycin solution, 10 mM HEPES buffer and 1 mM sodium pyruvate (all Sigma)). Coating, development and reading of ELISpot plates has been described previously [64]. A definition of positive responses was applied as: ≥50 SFU/ million, >4× background. For all assays 6 negative control wells (media only) and 2 positive control wells (10 µg/ml PHA (Sigma)) were used.

#### Cultured IFN-γ ELISpot assays

This assay has been previously described in Goonetilleke *et al.* [64]. In brief, short-term cell lines (STCL) were set up whereby 2×106 cell/ml of PBMCs were stimulated with a pool of peptides specific to the patient at 2 µg/ml in the presence of 25 ng/ml IL-7 (R&D Systems). Cells were cultured at 37°C, 5% CO_2_ for 10 days in RAB-10 (10% human AB serum, 86% RPMI 1640, 2 mM L-glutamine, 1× penicillin-streptomycin solution, 10 mM HEPES buffer and 1 mM sodium pyruvate (all Sigma)) and were stimulated with 1800 U/ml IL-2 (Novartis) on days 3 and 7. On day 10, the STCL were washed three times with PBS (Sigma) and rested for 30 hours in RAB-10 at 37°C, 5% CO_2_. On day 11, 4×104 cells/well were placed into an IFN-γ ELISpot as described above and stimulated by each individual peptide in triplicate at 2 µg/ml in the presence of RAB-10.

#### HLA typing

HLA typing was performed by T. Rostron (WIMM, Oxford UK) using the Sequence-Specific Primer (SSP) method adapted from Bunce *et al.* [65], that uses allele-specific primer combination in PCR amplification to provide absolute HLA resolution to 2-digits and high probability resolution to 4-digits.

### Intracellular cytokine assay

Cryopreserved peripheral blood mononuclear cells were thawed, rested overnight and stimulated for 6 hours at 37°C, 5% CO2 with 2 µg/ml of individual HIV-1 peptides representing the wild-type and escape sequences of the epitopic regions. The stimulation was conducted in presence of anti-CD28 mAb (1 mg/ml; L293; BD Biosciences), anti-CD49d mAb (1 mg/ml; L25; BD Biosciences), anti-CD107a-Alexa 680 (H4A3; BD Biosciences), 5 µg/ml Brefeldin A (Sigma) and 1 µg/ml Golgi Stop (Pharmingen). After stimulation, the PBMCs were stained with LIVE/DEAD Fixable Violet Dead Cell Stain (InVitrogen, Eugene, OR), anti-CD14-Cascade Blue (M5E2), anti-CD19-Cascade Blue (HIB 19) (to exclude non-viable cells), anti-CD4-Cy5.5-phycoerythrin (PE) (M-T477), anti-CD8-QD705 (RPA-T8), anti-CD27-Cy5-PE (M-T271), anti-CD57-QD605) (NK-1), anti-CD45RO-PE-Texas Red (all clones were BD Biosciences, but CD45RO BeckmanCoulter) for 20 minutes at room temperature. PBMC were then fixed and permeabilized with Cytofix/Cytoperm (Pharmingen). PBMC were stained with anti-CD3-Cy7-APC (SK7), anti-IFN-g-fluorescein isothiocyanate (FITC) (B27), anti-IL-2- Allophycocyanin (APC) (MQ1-17H12), anti-TNF-a-Cy7-PE (MAb11) and anti-MIP-1b-PE (D21-1351) (all BD Biosciences) for 45 min at 4°C. After washing and fixation samples were run on a custom built LSRII (BD Biosciences). A minimum of 300,000 total events was acquired using a custom made LSR II flow cytometer (BD Bioscience, San Jose, CA). Data analysis was performed using FlowJo 8.8.2 software (TreeStar), Pestle for background subtraction, and Spice for frequency analysis. (Pestle and Spice provided by Dr. M. Roederer Vaccine Research Center, NIH, Bethesda).

‘Memory’ CD8 T-cells are defined as live / lymphocytes / CD3 / CD8 / excluding the CD27hiCD45RO-negative population. Individual and total cytokine responses were considered as positive if the frequency of the total responding memory CD8+ subset was greater than 0.05% after background subtraction.

## Supporting Information

Figure S1 Highlighter plots (http://www.hiv.lanl.gov/content/sequence/HIGHLIGHT/highlighter.html) illustrating different patterns of hypermutation. Green stars show mutations consistent with Apobec hypermutation. Statistical tests of hypermutation were performed using Hypermut (http://www.hiv.lanl.gov/content/sequence/HYPERMUT/hypermut.html). (A) Sequences from a patient showing no evidence of hypermutation. (B) A patient with a single significantly hypermutated sequence (P = 0.003). (C) Sequences from a patient showing an overall elevated rate of hypermutation. No single sequence showed significant evidence of hypermutation; however, after compressing the mutations into a single sequence (using a tool available from the highlighter website) and re-running Hypermut with the compressed sequence, there was a significant elevation in the rate of G→A mutations in the Apobec sequence context (P = 9×10^−6^).
(1.46 MB TIF)

Table S1 Observerd mutations, sequence context within specific patients, and possible alternative explanations besides selection.
(0.09 MB XLS)
