# Supplementary figures and images for: HIV Evolution in Early Infection: Selection Pressures, Patterns of Insertion and Deletion, and the Impact of APOBEC
Source: PLoS Pathog. 2009 May 8;5(5):e1000414. doi: 10.1371/journal.ppat.1000414 (PMC2671846; doi:10.1371/journal.ppat.1000414)

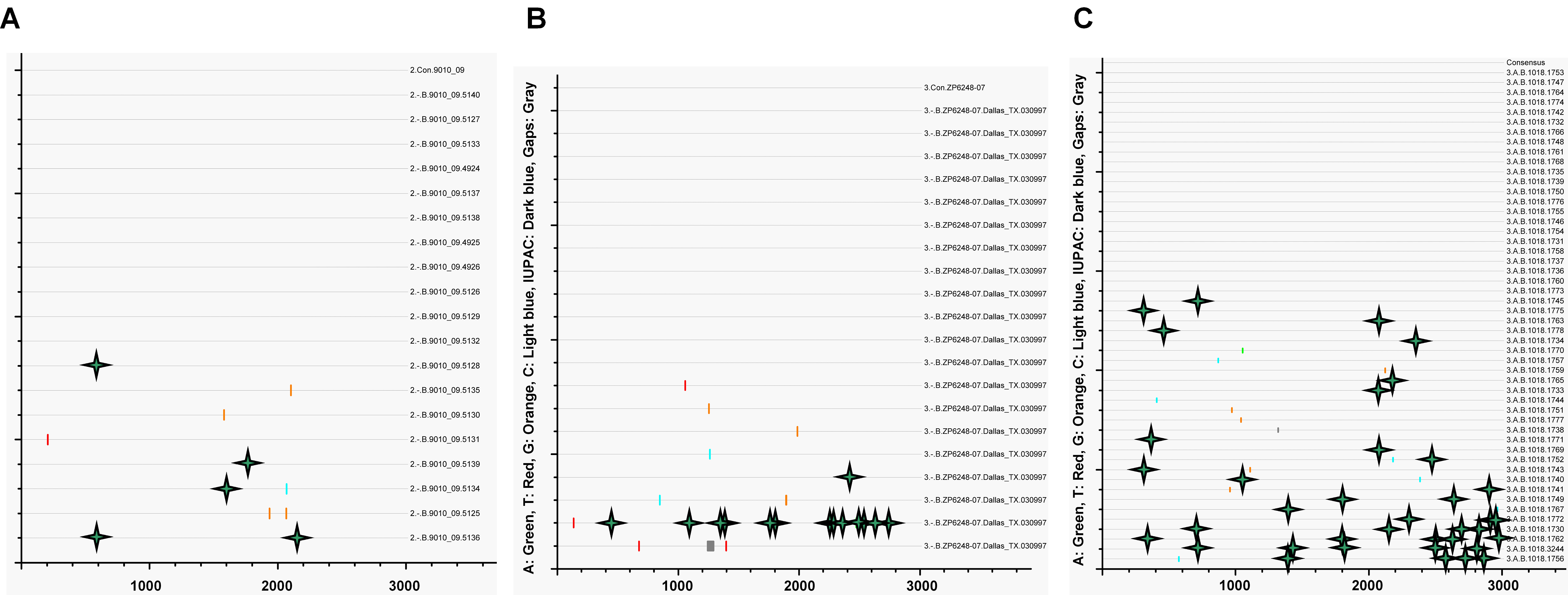

Supplement: Figure S1 — Highlighter plots (http://www.hiv.lanl.gov/content/sequence/HIGHLIGHT/highlighter.html) illustrating different patterns of hypermutation. Green stars show mutations consistent with Apobec hypermutation. Statistical tests of hypermutation were performed using Hypermut (http://www.hiv.lanl.gov/content/sequence/HYPERMUT/hypermut.html). (A) Sequences from a patient showing no evidence of hypermutation. (B) A patient with a single significantly hypermutated sequence (P = 0.003). (C) Sequences from a patient showing an overall elevated rate of hypermutation. No single sequence showed significant evidence of hypermutation; however, after compressing the mutations into a single sequence (using a tool available from the highlighter website) and re-running Hypermut with the compressed sequence, there was a significant elevation in the rate of G→A mutations in the Apobec sequence context (P = 9×10^−6^). (1.46 MB TIF) [file ppat.1000414.s001.tif]
